# Mechanisms of Ischemic Heart Injury

**DOI:** 10.3390/cells11091384

**Published:** 2022-04-19

**Authors:** Dominic P. Del Re

**Affiliations:** Department of Cell Biology and Molecular Medicine, Cardiovascular Research Institute, Rutgers New Jersey Medical School, Newark, NJ 07103, USA; delredo@njms.rutgers.edu

Ischemic heart disease is a leading cause of morbidity and mortality worldwide. Acute myocardial infarction (MI), commonly a result of coronary artery disease, elicits an enormous loss of cardiomyocytes and irreparable damage to heart muscle, leading to impaired cardiac function and, ultimately, heart failure. The most effective therapeutic intervention to treat patients suffering MI—typically percutaneous coronary intervention, thrombolytics, or coronary bypass grafting—provides timely reperfusion and limits the loss of viable myocardium. This rapid intervention, however, cannot prevent the additional tissue damage that results from the act of reperfusion itself, which contributes to injury. Importantly, an incomplete understanding of the cellular and molecular mechanisms underlying myocardial ischemia and reperfusion injury remains a significant barrier to improved therapeutics to mitigate cardiac damage.

This Special Issue is a collection of original and review articles that address multiple aspects related to ischemic heart injury and serves to extend and enhance our understanding of basic mechanisms underlying this phenomenon. Several of these topics, including mechanisms of regulated cell death, mitochondrial and protein quality control, inflammation, and the role of epigenetic and protein modifications in signal transduction, are highlighted.

The adult mammalian heart has limited regenerative capacity. Therefore, the preservation of viable myocardium in response to ischemic injury, an objective termed cardioprotection, is paramount and has been the aim of myriad preclinical studies and several clinical trials. The reversible modification of protein kinases, and their subsequent altered functionality, plays a fundamental role in signal transduction pathways that modulate cardioprotective responses in the ischemic heart. In this regard, Casin and Calvert provide a detailed overview of the role of a specific posttranslational modification, redox-sensitive cysteine oxidation, and its impact on select kinases that regulate cardioprotective signaling and ischemia/reperfusion injury [1]. The authors also discuss downstream signaling initiated by active kinases to promote cardiomyocyte survival, as well as highlight additional feedback mechanisms, whereby these kinases can modify the enzymes responsible for reactive oxygen species generation and modulation of the redox environment. Because reperfusion elicits a robust oxidative burst, a more complete understanding of how cysteine oxidation regulates critical cardioprotective kinases and their signaling is likely to play an important role in identifying novel therapeutic targets to limit injury.

The ability to improve mitochondrial health and maintain mitochondrial functionality has emerged as an important target for the preservation of viable myocardium in response to ischemic stress. This is facilitated by quality control mechanisms that include a cargo-specific form of autophagy, known as mitophagy, and its interaction with the mitochondrial dynamic processes, fission and fusion. Damaged mitochondria result from ischemia and reperfusion injury and can be selectively removed and degraded though active mitophagy, thereby maintaining the overall health of mitochondria and conferring protection of cardiomyocytes and heart tissue. The review article by Yu and Miyamoto discusses the importance of mitochondrial quality control and how mitochondrial dynamics are functionally linked to mitophagic mechanisms, resulting in the preservation of mitochondrial integrity and the prevention of cardiomyocyte death [2]. The authors also provide a detailed overview of stress-induced signaling that regulates mitochondrial quality control mechanisms, as well as mitochondria-mediated death pathways in the heart. Mitochondrial dysfunction is commonly observed in heart failure; therefore, a better understanding of the mechanisms that regulate mitochondrial quality control to prevent the accumulation of damaged mitochondria would likely serve to limit cardiomyocyte loss and thus remains an attractive potential therapeutic target.

Death of cardiomyocytes (i.e., cardiomyocyte dropout) is the fundamental driver of ischemic heart injury. There are currently at least 12 recognized programs of regulated cell death, and the number of those implicated in ischemic heart disease continues to expand [3]. Three of the most studied in a cardiac context include apoptosis, necrosis, and autophagy-related cell death. To this end, Kansakar et al. provide a detailed and comprehensive review of the contribution of each of these death routines in MI [4]. In addition, the authors delineate mechanistically how the epigenetic regulation of each death pathway is modulated through functional interactions with specific microRNAs. Furthermore, miRNAs as potential biomarkers for heart disease, and the therapeutic potential of leveraging miRNA-based approaches to treat heart disease, are also addressed.

A cell death program identified more recently, termed autosis, is thought to be caused by dysregulated autophagy, and has been reported to occur in the reperfused heart and mediate injury [5]. A new report from Nah et al. has identified the transcription factor Tfeb as a novel mediator of cardiomyocyte autosis during myocardial reperfusion [6]. The authors demonstrate that Tfeb is upregulated during reperfusion and promotes the expression of multiple autophagy-related genes, which likely facilitates the observed enhancement of autosis. Interestingly, the timing of Tfeb modulation appears to be critical for conferring cardioprotection, as downregulation of Tfeb prior to injury suppressed autosis but resulted in greater MI-induced tissue damage. Therefore, the authors postulate that inhibitory targeting of Tfeb during late reperfusion could provide cardiac benefit, and such an approach is warranted, as the modulation of autosis has not yet been explored therapeutically for the treatment of MI.

Following ischemic injury, the heart coordinates a multifaceted response in an attempt to prevent rupture and maintain pump function. This includes a highly orchestrated inflammatory response involving cardiomyocytes, cardiac fibroblasts, endothelial cells, and immune cells such as neutrophils, monocytes/macrophages, and lymphoid cells [7]. The cardiac fibroblast plays a key role in regulating inflammation and wound healing, and has therefore emerged as an attractive target for the modulation of adverse cardiac remodeling and progression to heart failure, as discussed in the review by Cakir et al. [8]. A detailed overview of the inflammatory and resolving phases of wound healing, differences between fibroblast subtypes, and the contribution of cardiac fibroblasts to post-infarct remodeling are addressed. Moreover, the translational aspects of therapeutic targeting of cardiac fibroblasts using both genetic and small molecule-based approaches are highlighted and discussed.

Original work from Iborra-Egea et al. leveraged an MI model in pigs and a systematic approach to examine and identify altered gene expression profiles, and their correlation with the progression of heart failure [9]. The authors performed microarray and comprehensive bioinformatics analyses using both infarcted and remote area heart tissue harvested from multiple timepoints after MI. Gene set enrichment analysis revealed altered processes, e.g., extracellular matrix remodeling, consistent with prior studies using small animal models. In addition, top gene candidates were predicted and prioritized as contributing to the observed remodeling processes found to be enriched post-MI. Importantly, the powerful tools employed here could be applied to additional omics-based studies to facilitate the identification of novel biomarkers and potential targets for therapeutic intervention.

The central contributions of increased reactive oxygen species and inflammation, altered metabolic substrate usage and mitochondrial dysfunction, and altered autophagy/mitophagy and mitochondrial dynamics in the progression of ischemic heart disease are examined in detail in the comprehensive review article from Schirone et al. [10]. Here, the authors carefully detail the underlying mechanisms and functional crosstalk between these processes and elucidate the similarities and differences between reperfused and non-reperfused MI. Implications for acute versus chronic phases of injury are also considered. Finally, translational insights are provided through highlighting an array of potential approaches to treat ischemic heart disease currently under evaluation, including cell- and exosome-based therapies, the use of bioengineered materials, and mitochondrial transplantation, among others.

In summary, this Special Issue highlights the diverse and complex mechanisms that underlie ischemic heart injury and the progression to heart failure. While much has been elucidated, a more complete understanding of these processes, and how they interact with one another to influence the extent of injury and subsequent pathology, is required to facilitate the field’s advancement toward novel therapeutic strategies to improve patient outcomes.
